# Retraction Note: The role of ascorbic acid combined exposure on Imidacloprid-induced oxidative stress and genotoxicity in Nile tilapia

**DOI:** 10.1038/s41598-024-71564-3

**Published:** 2024-09-10

**Authors:** Islam M. El-Garawani, Elsayed A. Khallaf, Alaa A. Alne-na-ei, Rehab G. Elgendy, Gaber A. M. Mersal, Hesham R. El-Seedi

**Affiliations:** 1 Zoology Department, Faculty of Science, Menoufia University, Shebin El-Kom, Menoufia 32512 Egypt; 2 Chemistry Department, College of Science, Taif University, P.O. Box 11099, 21944 Taif, Saudi Arabia; 3 Pharmacognosy Group, Department of Pharmaceutical Biosciences, BMC, Uppsala University, Box 591, 751 24 Uppsala, Sweden; 4 International Research Center for Food Nutrition and Safety, Jiangsu University, Zhenjiang, 212013 China; 5 Chemistry Department, Faculty of Science, Menoufia University, Shebin El-Kom, Menoufia 32512 Egypt

Retraction of: *Scientific Reports* 10.1038/s41598-021-94020-y, published online 19 July 2021

The Editors have retracted this Article.

After publication, concerns were raised regarding the data presented in Figures 4 and 6. Specifically:

- Different panels of Figures 4A–C appear to have duplicated features. These Panels represent tissues taken from animals which underwent different treatments.
- Different panels of Figure 6 appear to have duplicated features. These Panels represent tissues taken from animals which underwent different treatments.

The Authors provided raw data on request from the Editors. However, this was insufficient to satisfy the concerns raised. The Editors therefore no longer have confidence in the results and conclusions presented.

Islam M. El-Garawani, Rehab G. Elgendy, and Elsayed A. Khallaf do not agree to this retraction. Hesham R. El-Seedi, Alaa A. Alne-na-ei, and Gaber A. M. Mersal did not respond to correspondence from the Editor about this retraction.

